# Pediocins: The bacteriocins of Pediococci. Sources, production, properties and applications

**DOI:** 10.1186/1475-2859-8-3

**Published:** 2009-01-08

**Authors:** Maria Papagianni, Sofia Anastasiadou

**Affiliations:** 1 Department of Hygiene and Technology of Food of Animal Origin, School of Veterinary Medicine, Aristotle University of Thessaloniki, Thessaloniki 54006, Greece

## Abstract

Class IIa bacteriocins from lactic acid bacteria are small, cationic proteins with antilisterial activity. Within this class, the pediocins are those bacteriocins that share a highly conserved hydrophilic and charged N-terminal part harboring the consensus sequence -YGNGV- and a more variable hydrophobic and/or amphiphilic C-terminal part. Several pediocins have been isolated and characterized. Despite the structural similarities, their molecular weight varies, as well as their spectrum of antimicrobial activity. They exhibit important technological properties, e.g. thermostability and retaining of activity at a wide pH range, which along with the bactericidal action against Gram-positive food spoilage and pathogenic bacteria, make them an important class of biopreservatives. Much new information regarding the pediocins has emerged during the last years. In this review, we summarize and discuss all the available information regarding the sources of pediocins, the characteristics of their biosynthesis and production in fermentation systems, the characteristics of the known pediocin molecules, and their antibacterial action. The advances made by genetic engineering in improving the features of pediocins are also discussed, as well as their perspectives for future applications.

## Background

Peptides with antimicrobial properties (AMPs) are produced by eukaryotes and prokaryotes and serve as important components of their defense against microorganisms. Many bacteria are able to synthesize antimicrobial peptides. Those synthesized in the ribosomes, are generally referred to as bacteriocins (antibiotics are not included in the group since they are not ribosomally synthesized). The bacteriocins produced by Gram-negative bacteria are most often large proteins (many are larger that 20 kDa) and their inhibition spectrum is rather narrow, spanning to closely related species. Colicin V and the microcins, are exceptions as they are smaller than 10 kDa.

Gram-positive bacteria most often produce peptide bacteriocins smaller than 6 kDa. They are often cationic, amphiphilic, membrane-permealizing peptides, which in this manner resemble many of the AMPs produced by eukaryotes. They appear, however, to be effective in very low concentrations (often picomolar to nanomolar concentrations), although the action spectra are often narrow at these concentrations [1]. The bacteriocins produced by lactic acid bacteria (LAB) are divided into three main groups: the lantibiotics, which are modified bacteriocins (class I), the nonlantibiotics, which are heat-stable and unmodified (class II), and a group of large heat-labile bacteriocins (class III). Another group, known as class IV, is often included in classifications. Bacteriocins of class IV are complex molecules with lipid and carbohydrate moieties.

Most of the known ribosomally synthesized antimicrobial peptides produced by bacteria have been identified and studied during the last 20 years. Those produced by LAB, and in particular the lantibiotics, are the most extensively studied. Characteristics, among which antilisterial activity, increased specificity and effectiveness in very low concentrations, have long attracted the interest of the food sector for applications in food preservation. Nisin, the lantibiotic produced by *Lactococcus lactis *strains, is undoubtedly the most well-known, studied and characterized bacteriocin and the only one with widespread commercial use in most major food-producer countries. The success of nisin has led many research groups in searches for novel bacteriocin-producer strains and bacteriocins over the last years. This has resulted in a growing range of potential biopreservatives, with most promising the pediocins. These are AMPs produced by *Pediococcus *spp., which are categorized in the 2^nd ^class of bacteriocins from LAB, the known as "antilisterial" bacteriocins.

The potential applications of bacteriocins from LAB in the food and health care sectors have attracted the strong interest of academia and the industry resulting in an impressive amount of published research on their production, purification, genetics and applications. Since chemical preservatives are being continuously questioned with regard of safety, the use of LAB and their metabolites is generally accepted by consumers as something natural and health promoting. This offers a logical explanation for the non-reducing interest of the food scientists in the particular area and the expanding trend of applications of LAB in the food industry.

In this review, we will focus on the research and published literature on pediocins. Topics such as producer strains, characteristics of production in fermentation, structure of characterized pediocins, biochemical properties, antimicrobial spectra, and potential applications will be discussed in detail.

### The genus *Pediococcus*

*Pediococcus *is a genus of Gram-positive lactic acid bacteria, belonging to the family of *Lactobacillacea*. The genus *Pediococcus *consists of the following species: *P. acidilactici*, *P. pentosaceus*, *P. damnosus*, *P. parvulus*, *P. inopinatus*, *P. halophilus*, *P. dextrinicus*, and *P. urinaeequi *[2]. The often referred to as *P. cerevisiae*, is currently designated as *P. damnosus*, while strains formerly known as *P. cerevisiae *today are distributed among *P. damnosus*, *P. acidilactici *and *P. pentosaceus *[2-5]. Also, the taxonomic status of *P. halophilus *and *P. urinaeequi *remains uncertain [6,7]. *Pediococcus *spp. cells are spherical and arranged in tetrads, however, pairs are not uncommon in liquid cultures. They divide along two planes of symmetry, as do the other lactic acid cocci genera of *Tetragenococcus *and *Aerococcus*. They are facultative anaerobes, non motile and non sporulating [8]. The genus is paraphyletic and *P. dextrinicus *is only distantly related to the other species.

Pediococci are cultivated successfully in rich media [9]. Various species and strains differ in tolerance to oxygen, pH, temperature and NaCl [5]. They are homofermentative, although carbohydrate assimilation patterns and fermentation may differ among species and strains. Glucose is always fermented to racemic DL-lactate by the Embden-Meyerhof-Parnas (EMP) pathway [10]. Metabolic end products vary according to the conditions provided.

Among the known *Pediococcus *strains, *P. acidilactici*, *P. pentosaceus*, and *P. halophilus *are mostly associated with food fermentations. *P. acidilactici *and *P. pentosaceus*, take place in food fermentations either as indigenous microflora or in starters and both have been used in natural and controlled fermentations of vegetables and sausages [11,12]. Since pediococci typically are unable to ferment lactose [2], their applications in milk fermentations are restricted. There are however, a number of reports [13-15] which indicate that non starter and adjunct *Pediococcus *spp. impart desirable attributes to cheese suggesting that they may be good dairy starters if they possess the ability to utilize the particular sugar [16]. The ability of *P. acidilactici *and *P. pentosaceus *to produce antimicrobial peptides has attracted the interest for the use of either the cultures or their products as protective cultures or biopreservatives, respectively, in many foods. Both *P. acidilactici *and *P. pentosaceus *have also been used in silage fermentation, in the fermentation of dough and fruit juices, while several commercial probiotic feeds containing either species are currently available in the market.

*P. halophilus *(also known as *Tetragenococcus halophila*) plays an important role in the fermentation of miso and soy sause [11] and it is known as the soy *Pediococcus *[17,18]. The soy sauce mash or moromi has been the source for isolation of *P. halophilus *strains. The soy pediococci, salt-tolerant and homofermentative lactic acid bacteria, metabolize citrate and malate during lactic acid fermentation of soy sauce brewing. Citrate and malate are the acids that lactic acid bacteria most often encounter in their food environments and in the manufacture of fermented dairy products it is desirable that they are able to metabolize the two acids into especially, acetoin and diacetyl, both regarded as being favourable in enriching the flavours of cheese, butter and other products. Several strains however, have been described as non-citrate-metabolizing strains [19].

*P. damnosus *occurs in wine and cider and is found in brewery environments. It occurs as primary contaminant in pitching yeast and it is among the most prevalent spoilage microorganisms [20]. *P. damnosus *is sensitive to bacteriocins nisin of *Lactococcus lactis *[21] and pediocin AcH of *P. acidilactici *H [5], while it has been reported to produce a pediocin [22].

The various *Pediococcus *species exhibit different physiological characteristics which can be used for identification purposes. However, there are always strains within defined species that are different from the type strains. Various genetic tools have been used to discriminate between strains in the genus *Pediococcus*. These include the use of specific DNA target probes [23-25], ribotyping [26-28], total DNA-DNA hybridization [6], 16S rRNA gene sequencing [28,29]. Simpson et al. [30] studied the genomic diversity within the genus *Pediococcus *by randomly amplified polymorphic DNA PCR and pulse-field gel electrophoresis: specific DNA fragments within the *Not*I and *Asc*I macrorestriction patterns for each of the 33 examined strains from six species, were observed that allowed 27 of the 33 strains to be assigned to their proposed species. Following digestion with *Asc*I, all *P. parvulus *strains were characterized by two DNA fragments (220 kb and 700–800 kb). The exceptions correlated with those observed with both RAPD PCR primers and included three *P. damnosus *and two *P. pentosaceus *strains that grew at temperatures regarded as non permissive for their proposed strains but not for those with which they grouped.

Strains belonging to the species of *P. acidilactici *and *P. pentosaceus *have been separated by DNA-DNA homology, 16S rRNA, and mol% G+C in DNA techniques and by immunoassays. Bhunia and Johnson [31] have shown that the ELISA test with the monoclonal antibody Ped 2B2 can be used to differentiate between the two species, an approach that could find wider application in discriminating among closely related species.

Since the subject of this review is the pediocins-bacteriocins produced by *Pediococcus *spp. – the sections that follow will include information only on the pediocin producers: *P. acidilactici*, *P. pentosaceus *and *P. damnosus*.

### Pediocins

The Class II of unmodified bacteriocins is subdivided into the groups of the pediocin-like bacteriocins and the two-peptide bacteriocins. These are generally small (<5 kDa) and unmodified peptides. The pediocin-like bacteriocins (36–48 residues) are produced by many lactic acid bacteria and share a 40–60% amino acid sequence similarity [1]. The peptides of this group are known as "antilisterian" or "*Listeria*-active" peptides and they are characterized by a -Y-G-N-G-V-N- terminus. The hydrophilic N-terminal is well conserved. The N-terminal region of all pediocins currently identified contains two cysteines, joined by a disulfide bond, in a motif known as the "pediocin box": -Y-G-N-G-V-X_1_-C-X_2_-K/N-X_3_-X_4_-C-, with X_1–4 _representing polar uncharged or charged residues.

Pediocins are synthesized with a leader peptide attached which is removed by proteolytic processing, usually after a double glycine residue. Processing of pediocins has been reviewed for pediocin AcH and pediocin PA-1 [5]. Studies with pediocin AcH revealed that at the translation level it is synthesized as a biologically inactive peptide with 66 amino acid residues. It then undergoes a posttranslational modification, which includes the removal of a leader fragment of 18 amino acids from the N-terminal, to produce a 44 amino acid peptide that is biologically active [32]. Posttranslational modification is enzyme dependent and occurs at low pH at which activation of processing enzymes takes place [5,33]. Studies by other researchers [34,35] have shown that pediocin PA-1 undergoes a similar posttranslational processing.

### Pediocin producer strains

#### Pediococcus acidilactici

*P. acidilactici *strains are found in plants and milk. The optimum temperature for growth is 40°C. It is able however to grow at 50°C. A pH of 6.0 is regarded as the optimum for starting cultivation. During growth, the pH falls to levels as low as 3.6 [36]. Most strains ferment glucose, ribose, xylose, fructose and galactose to DL-lactate. A few strains are able to ferment lactose, sucrose and maltose [5]. Some strains have heme-requiring catalase, while all strains hydrolize arginine. Some strains have been found to produce pediocins. Pediocin optimum production conditions however, could differ from the optimum growth conditions [36]. *P. acidilactici *is used worldwide in fermentations of vegetables (e.g. sauerkraut), and meat-based products (e.g. dry sausages). Some vancomycin-resistant *P. acidilactici *strains have been found to be the responsible bacteria for repeated septicemia cases [37].

#### Pediococcus pentosaceus

The species share many characteristics in common with *P. acidilactici*, except a few: *P. pentosaceus *do not grow at 50°C, it withstands salt concentrations as high as 10%, while *P. acidilactici *do not, and the optimum temperature for growth is between 28 and 35°C. Most strains ferment glucose, ribose, galactose, arabinose, and fructose to DL-lactate. A few strains are able to ferment lactose, and xylose [5] and some strains are known to possess catalase activity [38,39]. *P. pentosaceus *have been implicated in a wide variety of fermentation processes, such as in the brewing industry and as starter cultures in sausage fermentations [40]. They also play important roles in silage fermentations and they are present in dairy products, vegetables and plant-based products (e.g. ripened cheese) [5,40,41]. Hexoses are metabolized by *P. pentosaceus *via the EMP (glycolytic) pathway, however, the oxidative metabolism, through aerobic reactions, and an active flavoprotein system were early investigated and presented by Dobrogosz and Stone [38]. Selective *P. pentosaceus *strains produce pediocins and have been the focus of much research with regard to food preservation. *P. pentosaceus*, appears in the literature to be more investigated than *P. acidilactici *from the physiology and genetics point of view. The full genome of *P. pentosaceus *ATCC 25745 has been sequenced [42,43] and is made up of 1,832,387 nucleotides organized in a circular manner. The genome has 1,755 protein encoding genes and 72 RNA genes and a 37.4% GC content. Plasmids associated with metabolism and bacteriocin synthesis have been isolated and characterized and a large number of transporter proteins are known today [44]. *P. pentosaceus *has been identified as an opportunistic pathogen [45,46], while it is considered as probiotic bacterium and an increasing number of probiotic products include it among other lactic acid bacteria.

#### Pediococcus damnosus

*P. damnosus *is phylogenetically distant from *P. acidilactici *and *P. pentosaceus *[47]. It does not grow at 35°C, the optimum temperature of *P. acidilactici *and *P. pentosaceus*, while its optimum temperature for growth is 22°C. The optimum pH for growth is 5.5. It does not grow at 4% NaCl, does not hydrolize arginine, arabinose, xylose, and lactose. Most strains ferment glucose, sucrose and galactose homofermentatively, and only some strains metabolize maltose and sucrose. As a beer and wine spoilage bacterium, has attracted research interest and recent publications report on the genetics [48-50], exopolysaccharide production [51] and bacteriocin production by *P. damnosus *[52-54].

### Growth and metabolism of pediocin producers *Pediococcus *spp

#### Growth conditions

The above described species are chemoorganotrophs. They require most amino acids and vitamins B, while *P. acidilactici *and *P. pentosaceus *require folic and and rivoflavin, respectively [9]. These two species do not have a growth requirement for NaCl [5]. They grow however, in media with 4% NaCl, while *P. halophilus *has a growth requirement for NaCl > 5%. Both *P. acidilactici *and *P. pentosaceus *require traces of Mn for growth [55].

Although categorized as facultative anaerobes or microaerophilic bacteria, both *P. acidilactici *and *P. pentosaceus*, grow rapidly aerobically and *P. halophilus *grows better aerobically [5]. Studies on growth and metabolism of *P. acidilactici *NRRL B 5627 performed in a stirred tank bioreactor under various dissolved oxygen tension levels (DOT) [36], show that aerobic, compared to semi-aerobic (60% DOT) and anaerobic, conditions led to higher biomass and lactate production levels and to lower pediocin levels. Similar results were obtained by the same group of Anastasiadou et al. [56] for the pediocin producer *P. pentosaceus *Mees 1934.

Information for *P. acidilactici *and *P. pentosaceus *on growth pHs and temperatures, as well as the ability to ferment various sugars has been given by Mundt et al. [57], Garvie [2] and Ray et al. [5]. Most strains are able to ferment glucose, fructose, galactose, mannose, cellobiose, arabinose, ribose, salicin, amygdalin, esculin, but not ferment sorbose, melibiose, inulin, starch, dextrin and sugar alcohols. Fermentation of other carbohydrates is strain dependent. Glucose has been found to have an inhibitory effect on growth of *P. acidilactici *cultures [58]. As mentioned earlier, glucose and other hexoses are metabolized by the EMP pathway. An active phosphoenolopyruvate:phosphotransferase system (PEP:PTS) is involved in the transfer of monosaccharides and data for a large number of transporter enzymes have been generated by genomic research and reported recently [43,44]. General PTS and sugar specific PTS enzymes of *P. pentosaceus *ATCC 25745 are known today. Information, however, on specific pathways and in depth research on sugar metabolism and transport mechanisms are still missing from the literature.

In general, there is great strain variation in the ability of *P. acidilactici *and *P. pentosaceus *to ferment carbohydrates such as lactose, sucrose, trealose, rhamnose and others [57]. Inability to ferment or low fermentation rates however, can be improved by genetic engineering strategies. This option could provide solutions to problems in the dairy industry. For example, pediococci (exceptions exist) are unable to ferment lactose but the development of lactose-positive *P. acidilactici *and *P. pentosaceus*, could be useful in replacement of cocci such as *Streptococcus thermophilus *in Italian starter blends [16]. Since these bacteria grow at 45°C and each has a long history of safe consumption in human food, construction of Lac^+ ^transformants would provide a solution to increased incidences of bacteriophage attacks on *S. thermophilus*. Lac^+ ^*P. acidilactici *and *P. pentosaceus *strains were constructed by transformation with the naturally occurring 35-kb *L. lactis *lactose plasmid, pPN-1, by Caldwell at et. [16]. The transformants were investigated for stability of the Lac^+ ^phenotype, the ability to acidify milk, and other important dairy properties. Results indicated that Lac^+ ^pediococci have potential as replacement cocci for *S. thermophilus *in starter cultures. The development of gene transfer systems for pediococci has been reported in some cases [16,59-61] and the uptake characteristics of genetically engineered pediococci for lactose and galactose have been investigated [61].

*P. pentosaceus *strains that possess a pseudocatalase system were found to be more efficient to glucose conversion to pyruvate under aerobic conditions [5]. The glycerol oxidizing system is inducible. A study of the end products of aerobic glycerol metabolism showed that glycerol was oxidized to the pyruvate level, producing lactate, acetate, acetoin and CO_2 _in a molar ratio of approximately 1:1:1:3 [38]. Acetoin can then be converted to diacetyl.

The lactate oxidation system of *P. pentosaceus *is inducible and helps the cells to derive energy from the oxidation of lactate to acetate [5]. Under aerobic conditions, L-(+)-lactate is oxidized to CO_2 _and acetate in equimolar amounts, while under anaerobic conditions, conversion of L-(+)-lactate to D-(-)-lactate takes place without production of acetate. Lactate oxidation by pediococci and the production of acetate and CO_2 _may contribute to the development of distinctive flavour and ripening of the cheddar cheese [5].

Citrate metabolism is inducible in the pediococci. Citrate metabolism has been studied in several strains of soy pediococci by Kanbe and Uchida [19]. It was observed that citrate-negative strains were missing the inducible citrate lyase [citrate (pro-3S)-lyase; EC 4.1.3.6], and pathways of citrate degradation in *P. halophilus *differed largely from those of other lactic acid bacteria. The main products from citrate were acetate and formate and *P. halophilus *did not produce acetoin or diacetyl. Formate production from citrate was greatly influenced (reduced) in the presence of glucose in the substrate.

*P. pentosaceus *possesses an inducible system for the metabolism of phenolic acids. The microorganism was found to display a substrate-inducible phenolic acid decarboxylase (PAD) activity on *p*-coumaric acid [62]. Barthelmebs et al. [62] showed that citrate metabolism is encoded by an autoregulated bicistronic operon which involves a new class of negative transcriptional regulator.

Pediococci do not produce extracellular proteases. Studies have shown however [13,14], that they have intracellular proteases, dipeptidases, and amino peptidases. The levels of the intracellular protein- and peptide-hydrolyzing enzymes may differ greatly with strains [5]. Pediococci probably do not produce extracellular lipases. They may contain lipases intracellularly, as was shown for *P. pentosaceus *but not for *P. acidilactici *[13]. The intracellular enzymes of pediococci have important roles in cheese ripening processes. The involvement of various intracellular enzymes, such as N-acetyl-muramidases, N-acetyl-glucosaminidases, l-alanine amidase and endopeptidases (peptidoglycan hydrolases) in the complex process of autolysis, that is important in fermentation processes, have only recently been studied with *P. acidilactici *and *P. pentosaceus *by Mora et al. [63]. The importance of autolysis of lactic acid bacteria in cheese ripening is well known and autolytic enzymes have been detected and characterized in many cases. Mora et al. [63] evaluated the autolytic phenotype in *P. acidilactici *and *P. pentosaceus *strains isolated from vegetable, meat and dairy sources. Peptidoglycan hydrolases of *Pediococcus *spp. were active in high salt concentrations and at different pHs, suggesting a potential role in salted meat and vegetable fermented products and in cheese ripening conditions when the pH is low and salt concentration is increasing. A strong effect of pediocin AcH/PA-1 on the level and rate of autolysis was identified with pediocin-sensitive strains.

### Conditions for pediocin production

#### Growth conditions

Pediocin production is greatly influenced by nutritional parameters, temperature, pH (initial and terminal pHs of fermentation broth), and aeration levels. Pediocin AcH, produced by *P. acidilactici *H, is an extensively studied and well-known pediocin [5,32,33,55]. The factors influencing the production of pediocin AcH have been reviewed by Ray [5,64]. A relatively simple broth (TGE) that contain only trypticase or tryptone, 1% glucose, 1% yeast extract, 1% Tween 80, 0.2% Mn^2+^, at initial pH of 6.5 resulted in higher pediocin production levels than MRS broth. Increase of trypticase, glucose, yeast extract to 2%, increased the yield by about a 10%. Production was highest with glucose, followed by sucrose, xylose, and galactose. Pediocin was not produced on arabinose, trehalose and raffinose, while growth and lactate production were very low with these carbohydrates.

Anastasiadou et al. [65] studied the influence of various nutritional papameters on pediocin production by *P. acidilactici *(pediocin SA-1, [36]) on a per cell basis. The study involved a direct plate bioassay procedure for rapid and quantifiable assessment of the carbon source and various salts. Solid-state cultivation of the microorganism was done on MRS-based media over 3- and 6-hours incubation periods. Glucose, sucrose, fructose, galactose and glycerol were evaluated. Glucose was found to be the optimal carbon source while glycerol exhibited the most suppressive effect. With glucose as the carbon source, addition of various salts in amounts used in liquid media commonly applied in the cultivation of pediococci, was assessed with respect to pediocin production per cell. Incorporation of NH_4_PO_4_, CaCl_2_, KH_2_PO_4 _into the medium resulted in an impressive suppression of pediocin production. Addition of MnSO_4_.H_2_O resulted in a significant increase in pediocin yield especially in the 6-hours assay. The direct plate assay proved to be a good pilot assay prior to conducting more intensive kinetic analysis in liquid cultivation.

Growth and metabolism of pediocin SA-1 producer *P. acidilactici *NRRL B5627 were studied under different aeration conditions in a stirred tank bioreactor using MRS broth [36]. Anaerobic cultivation produced very small amounts of pediocin. Fully aerobic conditions were again unfavourable for pediocin production. Dissolved oxygen tension maintained at 60% of saturation (semi-aerobic conditions) gave the highest pediocin concentration. The results pointed to a direct effect of dissolved oxygen on pediocin production with no correlative increase of biomass. This suggests that pediocin production is associated with an oxidative metabolic pathway. Semi-aerobic conditions were again the most favourable for pediocin SM-1 production by *P. pentosaceus *[56] with almost 4-fold higher pediocin yields compared to those obtained with other conditions. Although production of many bacteriocins produced by lactic acid bacteria has been studied under anaerobic conditions, there are certain cases like e.g. nisin [66,67] or amylovorin [68] in which an oxygen-enriched atmosphere enhanced production considerably.

Apart from the two studies by Anastasiadou et al. [36,56], literature information relative to pediocins and aeration levels applied during cultivation of the pediococci is, to the best of our knowledge, non existing. Most published studies have been carried out in flasks, without agitation and this applies also, to many other bacteriocins by LAB, with the exception of nisin. This may be due to a general perception of an anaerobic requirement of the LAB. Although anaerobic conditions could be the case for growth and lactate production, several studies on bacteriocins have made it clear that there is an oxygen requirement for successful production. Reliable studies (with cultivation performed in the controlled environment of bioreactors) include: the study by Cabo et al. [66], who reported that nisin A production from *L. lactis *at the maximum biomass point quadrupled when the oxygen saturation percentage increased from 50 to 100%; the work of Amiali et al. [67] with nisin Z, in which a requirement for 60% DOT was identified for increased production; the work of de Vuyst et al. [68] who reported on the oxygen demand for amylovorin biosyntheis from *Lactobacillus amylovorus *and pointed out the stimulation of bacteriocin production with primary metabolite kinetics under oxygen-enriched, and otherwise unfavourable for growth, conditions.

The optimum temperature for production of pediocin AcH from *P. acidilactici *H is 30°C [5]. Production of pediocin SA-1 from the strain *P. acidilactici *NRRL B5627 was also at 30°C [36]. A higher temperature was selected as the optimum for pediocin production for *P. acidilactici *F (a sausage isolate) [69]. Production of pediocins from *P. pentosaceus *strains was at 37°C for *P. pentosaceus *ACCEL [70], and *P. pentosaceus *L and S (isolated from pork meat) [71], 35°C for *P. pentosaceus *Pep1 (isolated from sausages) [72], 30°C for *P. pentosaceus *ST18 (isolated from boza drink) [73] and *P. pentosaceus *SM-1 (isolated from pork sausage) [56]. Pediocin PD-1 from *Pediococcus damnosus *NCFB1832 [54] is produced at 30°C.

In all published cases of pediocin production, culture pH was initially between 6.0 and 6.5. Then, it declines steadily to reach a value of 3.7 to 3.5 within approximately 25 hours. Large amounts of pediocin are secreted in the fermentation broth which is of significantly lower pH since the onset of fermentation and contains the produced in the meantime lactate [5,33-36,54]. The particular conditions are required for the secretion of active pediocin molecules, since posttranslational processing of the initially produced prepediocin takes place only at low pH at which the responsible enzymes are active [5]. The pH decline rate and the final pH reached in the cultures appear to be critical factors in pediocin production. It has been shown for pediocins produced by *P. acidilactici *that production displays primary metabolite kinetics depending on the pH decline rate [5,74-76].

### Production systems

In general, pediocin production displays primary metabolite kinetics with the rate of production paralleled the growth rate [5,36,56,58,74-76]. Pediocin AcH from *P. acidilactici *H, was detected in the fermentation broth in much higher amounts after the culture pH had reached 4.0 and growth entered the late exponential phase. Even after the cells had reached the stationary phase (18 hours), considerable amounts of pediocin were produced [5]. Anastasiadou et al. [36] presented kinetic studies of production of pediocin SA-1 from *P. acidilactici *and pediocin SM-1 from *P. pentosaceus *[56] at different dissolved oxygen levels. Under semi aerobic conditions, that supported the highest specific pediocin production rates, specific production rates followed the specific growth rates and production of both pediocins followed the trend of biomass production. In both cases, production ceased once the stationary phase of growth was reached. Maximum pediocin SA-1 levels were detected at 14 hours (160 AU/ml) and remained stable until 28 hours to decrease steadily beyond that point (127 AU/ml at 52 hours).

Pediocin production was mostly studied in batch culture and with synthetic media. Complex media have been applied in the studies of: Vazquez et al. [58] who used a waste medium for the production of pediocin from *P. acidilactici *NRRL B5627; Guerra and Pastrana [74], who cultivated *P. acidilactici *NRRL B5627 in whey; Vazquez et al. [77], who cultivated the same *Pediococcus *strain using waste protein sources from octopus; Nel et al. [78], who cultivated *P. damnosus *in acidic grape broth; Guerra and Pastrana [79], who used mussel-processing waste supplemented with glucose and five nitrogen sources; Vazquez et al. [80], who used peptones from autohydrolysed fish viscera; Guerra et al. [81,82], who cultivated *P. acidilactici *on whey and mussel-processing wastes in fed-batch culture.

Guerra et al. [81,82] reported increased pediocin yields, compared with batch fermentation, in fed-batch cultures with re-alkalization cycles. While growth was dependent on pH change, nitrogen and phosphorus availability and product inhibition (lactate, ethanol and butane-2,3-diol), pediocin production was dependent on both growth and the final pH reached in each re-alkalization period. The authors described in detail the time-courses of various fermentation parameters, e.g. biomass production, nutrient assimilation, pediocin activity and by-product formation and used the obtained data to construct mathematical models. Models that describe biomass and pediocin production were therefore presented for fed-batch cultures on whey and mussel-processing wastes. The developed models offered a better fit than the logistic or the Luedeking-Piret model and can be used to design feeding strategies for enhancing and controlling fed-batch pediocin production.

In a most recent publication, Guerra et al. [83] proceeded in modelling of the stress induced biphasic growth and pediocin production by *P. acidilactici *NRRL B5627 in re-alkalized fed-batch cultures. Using again whey or mussel processing wastes as substrates and employing the re-alkalization strategy, the authors observed and described a shift from homolactic to mixed acid metabolism and biphasic growth kinetics, which was attributed to a biphasic nitrogen metabolism. An unstructured mathematical model, based on the two phases on nitrogen consumption, was developed and expressed in terms of biomass, product accumulation and substrate utilization.

Pediocin production in continuous culture was reported by Cho et al. [84]. The authors presented a complex bioprocessing strategy for pediocin production involving continuous culture of an immobilized culture of *P. acidilactici *[85], carried out in a bioreactor packed with a fibrous matrix. Cell immobilization in the fibrous matrix was attained by natural attachment to fibres surface and entrapment in the void volume within the matrix. About 94% of cells were immobilized, the remaining remained suspended. Kinetics of fermentation and production of pediocin were investigated in dilution rates ranging from 0.63/day to 1.58/day and at pH values between 4.0 and 5.5. Maximum bacteriocin activity of 6400 AU/ml was obtained at dilution rate of at least 1.19/day, at pH controlled at 4.5. The system was operated for 3 months without contamination and clogging, or degeneration of the culture, exhibiting therefore very good long-term stability. The produced pediocin, designated as pediocin PO2, is a plasmid-encoded peptide with a molecular mass of approximately 4.6 kDa.

The effects of different dilution rates and pHs on pediocin 5 production by *P. acidilactici *UL5 were evaluated in continuous cultures of free and immobilized cells by Huang et al. [86]. A pH decrease from 7.0 to 5.0 led to a large increase in pediocin production (from 128 to 2048 AU/ml), at a dilution rate of 0.31/h in free cell cultures. The pH effect was not pronounced in immobilized cultures. At the optimum pH of 5.0, the dilution rate greatly influenced pediocin 5 activity in both free and immobilized cultures. Pediocin 5 production during continuous free cell culture decreased with time for all dilution rates tested, except 0.31/h, and average activity over 144 h cultures reached a maximal value of 4915 AU/ml at a dilution rate of 0.26/h. In immobilized culture, production was stable with time and increased with the dilution rate from 256 to 1024 AU/ml, in the range of 0.47 to 2.28/h.

From the above two works on pediocin production in continuous culture, it can be concluded that immobilized cultures offer the desired stability of fermentation for long periods but it cannot be concluded that increased overall productivity in such systems is secured. Unfortunately, the literature on the subject seems to be very limited. Recently, Naghmouchi et al. [87] reported the use of immobilized *P. acidilactici *UL5 cells in repeated-cycle batch cultures for pediocin PA-1 production. Cells were immobilized in locust bean gum gel beads and cultivated in MRS, supplemented with 1% glucose and whey permeate. The described system was stable and resulted in higher pediocin production levels and volumetric productivity at the end of 0.75- and 2-hour incubation cycles, compared with those of free cell cultures (productivity for pediocin: 5461 and 2048 AU/ml/h, for immobilized and free cells, respectively).

Pediocin production by *P. acidilactici *NRRL B5627 has also been investigated in solid-state fermentation. Vazquez Alvararez et al. [88] compared production characteristics in submerged fermentation and solid-state culture, carried out in polyurethane foam particles soaked in MRS or waste media (mussel processing waste) with various supplements. For the solid-state analysis, the cultures were treated by successive compression and refilling of tubular minireactors equipped with a piston, without the need for reinoculation. The method was found to be simple, easily controllable and reproducible. Culture productivity was maintained stable for long periods and yields were found to be superior compared to those from submerged culture.

### Characteristics of pediocins

#### Plasmid-associated production

Most Class IIa bacteriocin genes are plasmid located [89]. Graham and Mc Cay [90] were the first who reported that in *P. pentosaceus *FBB63, a plasmid of 15.7 kb is responsible for the pediocin phenotype. Association of plasmids of 19.4 and 8.3 kb in other *P. pentosaceus *strains was also reported by Daescel and Klaenhammer [91] and Hoover at al. [92], respectively.

In *P. acidilactici*, a relationship has early been reported between plasmid-associated bacteriocin production and carbohydrate fermentation patterns [5,59,92-94]. Although in various studies different plasmids are reported, e.g. pSMB 74, pSRQ 11, pPR 72, these plasmids are most likely the same [5], as the same molecule is pediocin AcH and pediocin PA-1, denoted today as pediocin AcH/PA-1 [5,25]. Applying a multilocus typing approach, Mora et al. [25] studied the genomic subpopulations within *P. acidilactici *(a wide selection of different environments of isolation of *P. acidilactici *was considered) and the relationship between pediocin AcH/PA-1 producing and non-producing strains and observed that three of the seven genotypes detected, showed relationships with pediocin AcH/PA-1 production and carbohydrate fermentation patterns. All pediocin-producing and sucrose-positive strains were grouped in genotype VII, melibiose-, sucrose- and raffinose-positive strains in genotype VI, and the arabinose-positive strains in genotype V. Plasmid profiles of *P. acidilactici *strains and plasmid-linked carbohydrate fermentation traits are given by Ray [5].

The class IIa bacteriocin genes are most often arranged in one or a few operons, and their organization has been reviewed by Ennahar et al. [95]. In the case of pediocin AcH/PA-1 the four genes needed for bacteriocin production and secretion are located in one operon [89]. The four genes are 1) the structural bacteriocin gene, encoding a prebacteriocin; 2) the immunity gene, encoding an immunity protein that protects the bacteriocin producer from its own bacteriocin; 3) the gene encoding the ABC transporter for secretion; and 4) a gene encoding a complementary protein of unknown function. The way these genes are organized in operons has been discussed in the review by Ennahar et al. [95].

#### Characteristics of pediocin molecules

The structure, as well as the structure-function relationship, of class IIa bacteriocins has been the subject of extensive research and has been most recently reviewed by Drider at al. [89]. Belonging to this class, pediocins are cationic peptides with similar primary structures. They contain two structural regions, a highly conserved N-terminal region, that harbors the consensus motif -YGNGV-, and a less conserved C-terminal region (residues 18 and on). The conserved -YGNGV- sequence was proposed initially as being responsible for the antilisterial activity of class IIa bacteriocins. Altering specifically residues within the motif resulted in dramatic loss of the activity: the activity of pediocin AcH/PA-1 was dramatically reduced by the Asn5-to-Lys mutation within the motif [96] and similarly the activity of carnobacteriocin B2 was reduced by replacing the Tyr3 with Phe [97]. More recent research however, indicated that some alterations with the -YGNGV- sequence can be tolerated [89].

The positively charged residues in class IIa bacteriocins are located mostly in the hydrophilic N-terminal region. It has been shown for pediocin AcH/PA-1 that electrostatic interactions and not the -YGNGV- motif, govern the binding of the pediocin and its fragments to phospholipids vesicles [98]. Lys11 and His12, that are part of the cationic patch in the N-terminal β-sheet-like region of pediocin AcH/PA-1, are of special importance for the electrostatic interactions and subsequent mutagenesis studies, in which charged residues in pediocin AcH/PA-1, and in sakacin P (another class IIa bacteriocin), were replaced by neutral residues confirmed earlier research [96,99,100].

The C-terminal region is important in determining the target cell specificity for class IIa bacteriocins [89]. This has been shown by combining N- and C-terminal regions from different class IIa bacteriocins (hybrid bacteriocins), which displayed target cell specificities similar to the the bacteriocins from which the C-terminal was derived [101]. Also, research carried out with pediocin AcH/PA-1 showed that by cleaving the area from residue 20 to residue 34, the bactericidal activity of the pediocin was inhibited [102]. This is an indication of a role for the C-terminal in recoginition of target cells.

Pediocin AcH/PA-1 from *P. acidilactici *PAC1.0 is the best-known pediocin so far. Its molecular weight is 16.5 kDa and it is plasmid-encoded by a 6.2 magadalton plasmid [93]. Other isolated and characterized pediocins from *P. acidilactici *spp. are: pediocin L50 from *P. acidilactici *L50, isolated from Spanish sausage, with M.W. of about 5.25 kDa [103]; pediocin AcM from *P. acidilactici *M, isolated from sausage, with M.W. of about 4.618 kDa [104]; pediocin F from *P. acidilactici *F, isolated from sausage, with M.W. of 4.46 kDa [69]; pediocin SA-1 from *P. acidilactici *NRLL B5627, with M.W. of 3.66 kDa [36]; and others with similar M.W. such as pediocin SJ-1 and pediocin N5p from *P. acidilactici *[105,106].

Isolated and characterized pediocins from *P. pentosaceus *spp. are: the designated as pentocins L and S from *P. pentosaceus *strains L and S isolated from pork meat, with M.W. of about 27 and 25 kDa, respectively [71]; a pediocin of 17.5 kDa M.W. produced by *P. pentosaceus *ACCEL, isolated from meat [70]; a two-peptide bacteriocin, pediocin ST18 from *P. pentosaceus *ST18, isolated from boza [73]; a 5 kDa pediocin from *P. pentosaceus *K23-2, isolated from Kimchi (a traditional Korean fermented vegetable) [107]; and the pediocin SM-1, a 5.37 kDa bacteriocin produced by *P. pentosaceus *SM-1, isolated from a Greek dry sausage [56].

Pediocin PD-1, with a M.W. of approximately 2.9 kDa is the only known pediocin that is produced by *P. damnosus*. It has been purified, characterized as molecule and studied with respect of its biochemical properties by Green et al. [22] and Bauer et al. [54].

### Antibacterial spectrum

In general, class IIa bacteriocins have a rather narrow spectrum of activity [89]. All class IIa bacteriocins are described as being active against *Listeria*. They are also active against some other Gram-positive pathogenic bacteria, such as *Clostridium *spp., and *Enterococcus *spp. Information on the spectra of antimicrobial activity is given for all isolated pediocins, however it is difficult to compare their potencies. Practical difficulties may come from the different ranges of indicators used or the different assays used for assessment of antimicrobial activity. Comparisons however, have made in some cases. Eijsink et al. [108] measured the activities of different bacteriocins, purified to homogeneity, against a large array of indicator strains. *L. monocytogenes *was among the most sensitive indicator microorganisms for the four bacteriocins examined. Pediocin AcH/PA-1 and enterocin A inhibited more strains than sakacin P and curvacin A. An extra C-terminal disulfide bridge was hypothesized as the responsible factor for the increased potency. Later, Fimland et al. [109] confirmed that in pediocin-like bacteriocins, the extra C-terminal disulfide bridge contributes to widening the antibacterial spectrum as well as to improving their potency at elevated temperatures.

The antimicrobial activity of pediocin AcH/PA-1 has been studied by Henderson et al. [34] and a list of strains inhibited by the pediocin was given. Elegado et al. [104] compared the antimicrobial activity spectrum of pediocin AcM, produced by *P. acidilactici *M, with that of pediocin AcH/PA-1. Pediocin AcM inhibited a large number of test bacteria including many species in the genera of *Lactococcus*, *Lactobacillus*, *Pediococcus*, *Leuconostoc *and *Enterococcus*, wider than that of the purified pediocin AcH/PA-1. It was very interesting that both pediocins, AcM and AcH/PA-1, inhibited the Gram-negative *Aeromonas hydrophila*. A later study [110] however, reported that pediocin AcH molecule did not adsorb onto *A. hydrophila*. Elegado et al. [104] commented that the produced halo in *A. hydrophila *may have been caused by either pediocin-resistant cells, or recovered cells after sublethal injury, or the inhibitory effect might have not been due to sole effect of the pediocin.

Next to pediocin AcH/PA-1, the pediocin produced by *P. acidilactici *NRRL B 5627 strain is another well-investigated pediocin with respect to its antimicrobial spectrum and conditions of production. Its molecule, however, has only recently been isolated and characterized by Anastasiadou et al. [36] and the pediocin has been designated as pediocin SA-1. Pediocin SA-1 is inhibitory to several food spoilage bacteria and food-borne pathogens. Table 1 gives the actimicrobial activity, in three degrees of sensitivity, of the purified pediocin SA-1 against a range of important for the food sector bacteria. It is not active against *Salmonella *spp., but compared with pediocin PD-1 (according to data given by Green et al. [22]) by *P. damnosus*, pediocin SA-1 appears to be significantly more effective against *Listeria *spp. Pediocin SA-1 exhibits intermediate inhibitory activity against other *P. acidilactici *strains and the phylogenetically close *P. pentosaceus*, unlike pediocin PD-1 by *P. damnosus *which showed no activity against these species [22] and small inhibitory activity against known bacteriocin producers *Lactobacillus sakei *CECT 906T, *Lb. plantarum *CECT 220, *L. lactis *ATCC 11454. Pediocin SA-1 was found to be very effective against the anaerobic *Clostridium sporogenes *and *C. thiaminolyticum*.

**Table 1:** Inhibitory spectrum of pediocin SA-1 produced by *Pediococcus acidilactici *NRRL B5627

Indicator organism	Cultivation conditions			Sensitivity *
	Medium	Incubation temperature	Aeration	
***Bacillus cereus *LMG13569**	BHI	30	Aerobic	+
***Clostridium sporogenes *NCTC533**	RCM	37	Anaerobic	+++
***Clostridium thiaminolyticum *ATCC15579**	RCM	37	Anaerobic	+++
***Enterococcus faecalis *NCTC8176**	MRS	37	Microaerophilic	++
***Lactobacillus brevis *ATCC8287**	MRS	37	Microaerophilic	+++
***Lactobacillus bulgaricus *LMG13551**	MRS	37	Microaerophilic	+++
***Lactobacillus casei *ATCC344**	MRS	37	Microaerophilic	+
***Lactobacillus curvatus *ATCC51436**	MRS	30	Microaerophilic	++
***Lactobacillus jensenii *ATCC25258**	MRS	37	Microaerophilic	+
***Lactobacillus plantarum *CECT220**	MRS	30	Microaerophilic	+
***Lactobacillus plantarum *NCAIM B 01133**	MRS	30	Microaerophilic	+
***Lactobacillus sakei *CECT906T**	MRS	30	Microaerophilic	+
***Lactococcus lactis *LM0230**	MRS	30	Microaerophilic	+
***Lactococcus lactis *ATCC11454**	MRS	30	Microaerophilic	+
***Lactococcus lactis *IL1403**	MRS	30	Microaerophilic	+
***Lactococcus lactis *spp. *cremoris *MC1363**	MRS	30	Microaerophilic	+
***Leuconostoc mesenteroides *spp. *crem*ATCC19254**	MRS	25	Microaerophilic	+++
***Listeria inocua *ATCC BAA-680D**	BHI	30	Microaerophilic	+++
***Listeria monocytogenes *ATCC19111**	BHI	30	Microaerophilic	+++
***Micrococcus flavus *ATCC400**	NB	30	Aerobic	+++
***Micrococcus luteus *CECT241**	NB	30	Aerobic	+++
***Pediococcus acidilactici *ATCC25740**	MRS	30	Microaerophilic	++
***Pediococcus pentosaceus *ATCC 33316**	MRS	30	Microaerophilic	++
***Pediococcus pentosaceus *LMG13560**	MRS	30	Microaerophilic	++
***Pediococcus pentosaceus *NRRL B14009**	MRS	30	Microaerophilic	++
***Salmonella enteritidis *ATCC13076**	SS	25	Microaerophilic	-
***Staphylococcus carnosus *LMG13564**	BHI	37	Microaerophilic	++

*+, sensitivity to pediocin SA-1 given at three degrees +, ++, +++

-, resistant to pediocin SA-1

Another recently isolated and characterized pediocin, the pediocin SM-1 from *P. pentosaceus *SM-1 (a Mees 1934 strain) studied by the same group of researchers as pediocin PA-1[56] and their spectra of activities were compared using the same set of indicator microorganisms. Pediocin SM-1 was found also to be very effective against *L. monocytogenes *and *L. inocua*, as well as against the anaerobic *C. sporogenes *and *C. thiaminolyticum*. A smaller degree of activity was shown against several lactic acid bacteria species, among which *Pediococcus *spp. Compared with pediocin SA-1, it appears to be more active against LAB and *Listeria *spp., and compared with the data given by Green et al. [22] for pediocin PD-1 by *P. damnosus*, it appears to be significantly more effective against *Listeria*. Pediocin SM-1 is not active against the Gram-negative *Salmonella *spp.

The antimicrobial activity against a wide range of Gram-positive bacteria and the minimum inhibition concentrations of pediocin L50, produced by *P. acidilactici *L50, have been presented by Cintas et al. [103]. Its inhibitory spectrum was found to be broad, although resistant lactobacilli were found, and it was very effective against *L. monocytogenes*. The antimicrobial activity of pediocin PD-1 against a wide range of bacteria has been discussed by Green et al. [22].

Although there is high structural similarity in pediocins, and within the class IIa of bacteriocins, sensitivities of target-bacteria may vary markedly. There should be a number of reasons for this, as for example the existence of cell surface structures with which the bacteriocin interacts, or the composition of cell membrane, or the classical antibacterial assays which are not very sensitive, and many others. Further information on the subject can be found in the recent and important review by Drider et al. [89] on the "continuing story" of class IIa bacteriocins.

### Mode of action

The cytoplasmic membrane of Gram-positive bacteria is the target of pediocins [1]. All the class IIa bacteriocins whose modes of action have been studied permealize the cytoplasmic membrane through pore formation by insertion of the C-terminal regions into the membrane [89]. However, the specific role of the YGNGV motif of the pediocins has not clarified yet. Pediocins are bactericidal to sensitive Gram-positive bacteria [5]. Being hydrophobic molecules, they destabilize the cytoplasmic membrane when they come in contact with it. This action includes loss of the permeability barrier and loss of the membrane potential, which, in strains that possess an autolytic system, result in cell lysis.

The mode of action of pediocin AcH/PA-1 has been studied by Bhunia et al. [110], Motlagh et al. [111] and Ray [5,64]. Loss of intacellular K^+^, entrance of lactose from the medium inside the cells and cell lysis of some strains, indicated the destabilization of the membrane functions by the pediocin. Pediocin AcH/PA-1 was found to bind to receptors on the cell surface of both sensitive and resistant cells to it [110]. Destabilization of the membrane occurred only in sensitive cells because of some types of conformation alterations which took place only to them and impaired the permeability of the membrane. The resistance of the producer strain *P. acidilactici *H to pediocin AcH/PA-1, is controlled by an immunity protein encoded by the specific immunity gene [5]. Gram-negative bacteria do not adsorb the pediocin and this may be the reason of their resistance. Injured, by sublethal stresses, Gram-negative bacteria became sensitive to pediocin. It was postulated that pediocin AcH/PA-1 enters the cells through the damaged outer membrane, comes in contact with the inner membrane and destabilizes its functions and kills the cells [5,64]. Spores also of Gram-positive bacteria do not adsorb the pediocin AcH/PA-1 [5]. Following germination and outgrowth, the sensitive cells adsorb the pediocin and are killed.

More purified pediocins have been examined for their mode of action in the works of Green et al. [22] and Bauer et al. [54] for pediocin PD-1 produced by *P. damnosus*, and the works of Anastasiadou et al. [36,56] for pediocins SA-1 from *P. acidilactici *and SM-1 from *P. pentosaceus*. Using the indicator bacterium *Oenococcus oeni*, Bauer et al. [54] concluded that pediocin PD-1 acts on its cytoplasmic membrane and the antimicrobial activity is due to the generation of pores in the membrane. The ability of pediocin PD-1 to form pores in sensitive cells of *O. oeni*, as observed by K^+ ^loss was found to be pH dependent and increased when the extracellular pH was reduced from 7.0 to 5.0. Although the rate of pediocin induced K^+ ^loss was the highest at pH 5.0, the initial rate of K^+ ^loss was the highest at pH 6.0. This suggest that the DpH driving force contributes to the action of the pediocin, as it has been also observed with the lantibiotic nisin from *L. lactis *[112], and a synergistic effect between pediocin PD-1 and pH is possible. In the presence of 10 mM gadolinium (Gd3^+^), pediocin PD-1 did not affect cells of *O. oeni*. This suggests that the mode of action of the pediocin relies on a net negatively charged cell surface. Following detailed investigations on the K^+ ^efflux and comparisons with nisin, Bauer et al. [54] proposed that cell death is a result of membrane disruption and a rather slow process of loss of metabolites rather than an immediate loss, as happens with nisin. Studies with treatment of sensitive *O. oeni *cells with cations, led to the postulation that pediocin PD-1 may also inhibit cell wall biosynthesis, which leads to cell lysis. Anastasiadou et al. [36,56] observed a bactericidal mode of action in studies with purified pediocins SA-1 and SM-1 and indicator cells of *Micrococcus luteus *at mid-logarithmic phase of growth. The decrease in colony forming units of plated *M. luteus *from 10^8 ^to 0 with 4 hours, was accompanied by unchanged turbidity at OD 600 nm (no cell lysis). Cell suspensions of treated cells were examined for the presence of DNA and for increase of protein levels and in both cases were negative. Similar were the findings with pediocin PD-1 and *O. oeni *cells, as reported by Green et al. [22].

All class IIa bacteriocins are believed to bind to a chiral receptor in cell membranes and create a pore that depolarizes the target cell. The exact nature of the bacteriocin-receptor interaction is not yet understood, but it appears to be mediated by the membrane bound proteins mptC and/or mptD [113]. In their most recent work, Derksen et al. [113] proceeded in synthesis of class IIa bacteriocins (pediocin PA-1 and leucocinA) mutants and analogues, to investigate the structure-function relationship and to obtain further information on the peptide-receptor complex. Their results were consistent with the idea that the selectivity and activity of class IIa bacteriocins is dependent on their entire sequence and the overall three-dimensional structure preferred in membranes.

The factors affecting the efficacy of the various pediocins against sensitive target-cells are less investigated. Bauer et al. [54] presented results that show the activity of pediocin PD-1 to be dependent on the growth temperature of the indicator bacterium *O. oeni*. This was attributed to membrane fluidity changes and alteration of its lipid and protein contents that affect the resistance of the membrane to pore formation. The authors stress the need for further studies on the efficacy of antimicrobial peptides in general, under a range of growth phases and conditions before making generalized conclusions. When food is in concern, it is important to distinguish among the various growth phases of the target-cells in order to assure effective control and preservation. In this respect, characteristic is the study by Todorov and Dicks [73] with the antimicrobial effect of pediocin ST18 on *L. innocua *F. Addition of bacteriocin containing cell-free filtrate (3200 AU/ml) to logarithmic-phase cells resulted in growth inhibition after 1 hour that completed in 2 hours. Addition of the same activity level of pediocin to stationary-phase cells of *L. innocua *resulted in no growth inhibition.

### Physicochemical and biochemical properties of pediocins

The known pediocins from *P. acidilactici*, *P. pentosaceus *and *P. damnosus *strains are mostly small, hydrophobic proteins. Their bactericidal action is stable to heat treatment, sometimes even at sterilization temperatures, as well as to cold treatment, even at -80°C. Their activity is retained at a wide pH range. They are sensitive to most proteases. These characteristics are common in a number of pediocins as shown with pediocin AcH/PA-1 [5], pediocin ST18 [73], pediocin AcM [104], pediocin L50 [103], pediocin PD-1 [22], pediocin F [69], the pediocins produced by *P. pentosceus *Pep1 [72] and *P. pentosaceus *K23-2 [107].

Table 2 summarizes some of the properties of isolated pediocins, in the cases the particular characteristics were examined and allow comparisons. The obvious differences, as shown in Table 2, may result in differences in the way of action and may explain the differences found in the inhibitory spectra of various pediocins. In overall, it can be concluded that pediocins have important technological properties for being considered as mild antimicrobials and biopreservatives.

**Table 2:** Effects of heat, pH and proteolytic enzyme treatments on the antimicrobial activity of various isolated pediocins

Treatment*										
	Temperature		pH		Proteolytic enzymes					
Pediocin	100°C/60 min	121°C/60 min	2–10	4–7	Pepsin	Papain	Trypsin	α-chymotrypsin	Proteinase K	**Reference**
SM-1	+	+			-	-	-	-	-	Anastasiadou et al. 2008
	+	+	+	+	-	ND	ND	ND	-	
pK23-2				+						Shin et al. 2008
			+							
SA-1	+	+	+	+	+	+	+	+	-	Anastasiadou et al. 2007
ACCEL	+	±	+	+	-	-	-	-	-	Wu et al. 2004
PD-1	+	+	+	+	+	+	+	+	-	Green et al.1997
SJ-1	+	+	-	+	ND	-	-	-	-	Schved et al. 1993
N5p	+	+	-	+	ND	ND	ND	ND	ND	Strässer et al. 1995
AcH	+	+	+	+	ND	-	-	-	-	Bhunia et al. 1988
PA-1	+	±	+	+	-	-	ND	ND	ND	Gonzales and Kunka 1987

* +, Activity; -, Absence of any activity; ND, not determined.

Pediocin AcH/PA-1 was the first to be studied with respect to its biochemical properties [5]. The recently isolated and characterized pediocin SA-1 from *P. acidilactici *NRRL B5627 [36] was also found to be a very stable pediocin at various conditions. The purified pediocin SA-1 is heat stable for up to 60 min at 121°C. Storage for 4 weeks at -80, -20, 4 and 30°C did not affect its antimicrobial activity. This was not impaired even following incubation at 30°C for 1 week at pH values ranging between 3.0 to 12.0. No antimicrobial activity was detected however, after 30 min of incubation in buffers of pH 2.0, 13.0 and 14.0. Pediocin SA-1 was found to be resistant to treatment with trypsin, α-chymotrypsin, pepsin and papain, but not to proteinase K. Pediocin SM-1 from *P. pentosaceus *SM-1 [56] was also found to be inactivated by the above enzymes. The purified pediocin ACCEL, produced by *P. pentosaceus *ACCEL [70], was inactivated by proteolytic treatment and remained stable with the narrower pH range of 2.0 to 6.0 and at lower temperatures of <100°C. However, more of 80% of its activity was remained even after 15 min of heating at 121°C, at pH 2.0–4.0. The also recently isolated pediocin from *P. pentosaceus *K23-2 [107] was found to be very heat-stable, as its activity remained unchanged following 15 min of incubation at 121°C.

Pediocins, as all class IIa bacteriocins, are essentially unstructured in water (have random coil structures) and assume a defined conformation only in hydrophobic environments or in solvents [89,113,114]. Various environmental factors, e.g. temperature, pH and others, affect their defined structure and their antimicrobial activity. Kaur et al. [114] studied the effects of temperature on antimicrobial activity and on structure of the C-terminal amphipathic α helix as a receptor-binding region. They reported that at elevated temperatures pediocin PA-1 maintains its overall structure, whereas peptides without a second C-terminal disulfide bond, such as sakacin P, curvacin A, enterocin P, experience partial disruption of the helical section. Pediocin PA-1 and a chemically synthesized mutant of it, in which methionine was replaced by norleucine (for enhanced stability toward aerobic oxidation), were found to be equally active at different temperatures, whereas the peptides that lack the second disulfide bond in the C-terminal were 30–50 times less antimicrobially potent at 37°C than at 25°C. The structural changes in the helical region observed (CD spectroscopy was employed) at elevated temperatures account for the loss of activity of these peptides. The presence of the C-terminal disulphide bond in pediocin PA-1 helps in maintaining the tertiary structure of the peptide at elevated temperatures, while in the absence of it the peptides experience a significant structural peprturbation in the amphipathic helix region (loss of the helical content). The hydrophobic residues of the C-terminal helical domain orient themshelves on one side of the helix, facilitating this way a specific interaction with the receptor protein (probably a region of the mannose specific PTS subunit IID) on the target cell. The study of Kaur et al. [114] showed that the α helix in the C-terminal of the pediocins is critical for activity and helped significantly in the understanding of the structure-function relationship.

### Applications and perspectives

Biopreservation systems, such as bacteriocinogenic cultures and/or their bacteriocins, have received increasing attention in recent years [115]. Pure or mixed cultures of bacteriocin-producer lactic acid bacteria, including the pediococci, are marketed today as protective cultures against common food spoilage bacteria and pathogens. Application of such cultures may lead to improvements in food quality and sensory attributes through controlling adventitious flora and inducing cell lysis [116]. Although there are commercial enzymes that can be used to carry out cell lysis, their controlled application is difficult and they can be expensive, while bacteriocin-producer cultures can perform the process in a milder and less expensive way and with overall improvements of flavour and quality because of the growth of the cultures themselves and the production of other metabolites at the same time. Danisco have formulated a freeze-dried *P. acidilactici *culture, marketed as CHOOZIT™ Lyo. Flav 43, which is suggested for use in cheddar cheese and semi-hard cheeses as an adjunct that "accelerates and enhances strong and sweet flavour compounds, due to the production of bacteriocins".

The number of scientific publications on the use of bacteriocins for food preservation is currently steadily increasing. The subject is covered by recent excellent reviews, such as those by Deegan et al. [115] and Guinane et al. [116]. Although many bacteriocins exhibit significant antimicrobial and other technologically important qualities, the use of a bacteriocin alone in a food cannot ensure sufficient safety. Gram-negative bacteria do not represent target cells for bacteriocins as they are protected by an outer membrane. It is therefore necessary that bacteriocins are used in combination with other preservation methods (e.g. other antimicrobials, or organic acids) and according to Deegan et al. [115] it may emerge that industrially they will be applied as a final hurdle in a food system following others that already exist and eliminate the non-targets to bacteriocins pathogens and spoilers. Until recently, nisin was the only bacteriocin marketed as food biopreservative. Recently, a pediocin by *P. acidilactici *containing formulation is marketed under the commercial name Alta 2341^®^. Although research results regarding the efficiency of bacteriocins as biopreservatives are remarkable and promising, there is substantial reluctance by the industry to commit financially in developing commercial bacteriocin preparations. This is because of the costly production (low production rates, unstable products and expensive downstream processing) and difficulties that can arise from legislation. The costly production can be counteracted by suitable bioprocessing strategies designed for increased yields and by genetic engineering approaches.

Genetic engineering has been applied with *Pediococcus *spp. and pediocins in studies of the physiology of the producer-microorganisms, the complex structure-function relationship and the specificity of the pediocins, or for improvements in utilization of certain substrates, e.g. lactose, or even the construction of hybrid bacteriocins with improved stability and other properties [16,101,102,113,114,117,118]. The most desired characteristic when a pediocin is to be used as a food additive is its stability in the complex environment of food. Several of the pediocin-like bacteriocins contain methionine residues whose sulfur atom may be oxidized, resulting in destabilization of the bacteriocin. Johnsen et al. [119] focused on the methionine residue of the pediocin PA-1 and constructed variants in which the Met13 residue was replaced by Ala, Leu, Ile, and Asp. Replacing the Met13 with the first three, protected the peptide from oxidation and had only minor effects on antimicrobial activity, while replacement by Asp resulted in a marked decrease in potency against all indicator strains used. Therefore, making pediocin PA-1 more stable by replacing the methionine with another hydrophobic residue and retaining its activity is an important step in developing pediocin PA-1 into a useful food additive.

Research in the topic of the genetic engineering of pediocins is rather limited. Continued research may lead to pediocins with increased stability and enhanced features, or extension of the antimicrobial spectrum to Gram-negative bacteria. The engineering strategies may yield characteristics that could be very rewarding from a food safety and an economic point of view, however, they can be counterproductive from the marketing point of view, since they will yield products-pediocins made by genetic engineering with all the associated consumer concerns.

Food application of pediocins can provide a good alternative means in protecting food against foodborne pathogens. As products of lactic acid bacteria, they provide natural means of preservation and can be accepted by the consumers in the way nisin became accepted. As the trend of consumption of minimal processed and preserved foods is increasing, use of pediocins by the food industry could offer solutions and provide alternatives of conventional preservation means. Being mild antimicrobials, pediocins are also expected in the future to find more applications in both human and veterinary medicine. The growing problem of bacterial resistance to antibiotics could be faced by using antibiotic complements of bacteriocins, as studies have shown for nisin [119-121].

## Competing interests

The authors declare that they have no competing interests.

## Authors' contributions

MP drafted the manuscript. SA contributed additional content throughout the article. All authors have read and approved the final manuscript.

## References

[B1] Papagianni M (2003). Ribosomally synthesized peptides with antimicrobial properties: biosynthesis, structure, function, and applications. Biotechnol Adv.

[B2] Garvie EI (1986). Genus *Pediococcus *claussen 1903. Bergey's Manual of Systemic Bacteriology Sneath, PHA.

[B3] Gherna R, Pienta P (1989). American Type Culture Collection: Catalogue of Bacteria and Phages.

[B4] Kitahara K, Buchanan RE, Gibbons NE (1974). Genus *Pediococcus balcke *1884, 247. Bergey's Manual of Determinative Bacteriology.

[B5] Ray B, Hui YH, Khachatourians G (1995). *Pediococcus *in Fermented Foods. Food Biotechnology: Microorganisms.

[B6] Dallaglio F, Trovatelli LD, Sarra PG (1981). DNA-DNA homology among representative strains of the genus *Pediococcus*. Zentbl Bakteriol Mikrobiol Hyg.

[B7] Collins MD, Williams AM, Wallbanks S (1990). The phylogeny of *Aerococcus *and *Pediococcus *as determined by 16S rRNA sequence analysis: description of *Tetragenococcus *gen. nov. FEMS Microbiol Lett.

[B8] Schlegel HG (1993). General Microbiology. English translation.

[B9] Atlas RM (2004). Handbook of microbiological media.

[B10] Gasson MJ, DeVos WM, (editors) (1994). Genetics and biotechnology of lactic acid bacteria.

[B11] Wood BJB (1997). Microbiology of Fermented Foods.

[B12] Knorr D (1998). Technological aspects related to microorganisms in functional foods. Trends Food Sci Technol.

[B13] Bhowmik T, Marth EH (1990). Role of *Micrococcus *and *Pediococcus *species in cheese ripening: a review. J Dairy Sci.

[B14] Bhowmik T, Riesterer R, van Boekel MAJS, Marth EH (1990). Characteristics of low-fat cheddar cheese made with added Micrococcus or *Pediococcus *species. Milchwissenschaft.

[B15] Reinbold GW, Reddy MS (1978). Preparation of pizza cheese. US patent 4,085,228.

[B16] Caldwell SL, McMahon DJ, Oberg CJ, Broadbent JR (1996). Development and characterization of lactose-positive *Pediococcus *species for milk fermentation. Appl Environ Microbiol.

[B17] Nakagawa A, Kitahara K (1959). Taxonomic studies on the genus *Pediococcus*. J Gen Microbiol.

[B18] Sakaguchi K (1958). Studies on the activities of bacteria in soy sauce brewing. Part 3. Taxonomic studies on *Pediococcus sojae *nov. sp., the soy sauce lactic acid bacteria. Bull Agric Chem Soc Jpn.

[B19] Kanbe C, Uchida K (1987). Citrate metabolism by *Pediococcus halophilus*. Appl Environ Microbiol.

[B20] Priest FG, Priest FG, Campbell I (1996). Gram-positive brewery bacteria. Brewing Microbiology.

[B21] Delves-Broughton J (1990). Nisin and its uses as a food preservation. Food Technol.

[B22] Green G, Dicks LMT, Bruggeman G, Vandamme EJ, Chikindas ML (1997). Pediocin PD-1, a bactericidal antimicrobial peptide from *Pediococcus damnosus *NCFB 1832. J Appl Microbiol.

[B23] Mora D, Fortina MG, Parini C, Manachini PL (1997). Identification of *Pediococcus acidilactici *and *Pediococcus pentosaceus *based on 16S rRNA and *ldh*D-targeted multiplex PCR analysis. FEMS Microbiol Lett.

[B24] Mora D, Parini C, Fortina MG, Manachini PL (1998). Discrimination among pediocin AcH/PA-1 producer strains by comparison of *ped *B and *ped*D amplified genes and multiplex PCR assay. Syst Appl Microbiol.

[B25] Mora D, Fortina MG, Parini C, Daffonchio D, Manachini PL (2000). Genomic sub-populations within the species *Pediococcus acidilactici *detected multilocus typing analysis: relationships between pediocin AcH/PA-1 producing and non-producing strains. Microbiology.

[B26] Jager K, Harlander S (1992). Characterization of a bacteriocin from *Pediococcus acidilactici *PC and comparison of bacteriocin-producing strains using molecular typing procedures. Appl Microbiol Biotechnol.

[B27] Satokari R, Mattila-Sandholm T, Suihko ML (2000). Identification of pediococci by ribotyping. J Appl Microbiol.

[B28] Barney M, Volgyi A, Navarro A, Ryder D (2002). Riboprinting and 16S rRNA gene sequencing for identification of brewery *Pediococcus *isolates. Appl Environ Microbiol.

[B29] Omar NB, Ampe F, Raimbault M, Guyot JP, Tailliez P (2000). Molecular diversity of lactic acid bacteria from cassava sour starch (Colombia). Syst Appl Microbiol.

[B30] Simpson PJ, Stanton C, Fitzerald GF, Ross RP (2002). Genomic diversity within the genus *Pediococcus *as revealed by randomly amplified polymorphic DNA PCR and pulsed-field gel electrophoresis. Appl Environ Microbiol.

[B31] Bhunia AK, Johnson MG (1992). Monoclonal antibody colony immunoblot method specific for isolation of *Pediococcus acidilactici *from foods and correlation with pediocin (bacteriocin) production. Appl Environ Microbiol.

[B32] Motlagh AM, Bhunia AK, Szostek F, Hansen T, Johnson MC, Ray B (1992). Nucleotide and amino acid sequence of pap-gene (pediocin AcH production) in *Pediococcus acidilactici *H. Lett Appl Microbiol.

[B33] Johnson MC, Hanlin MB, Ray B Low pH and lactate are necessary for conversion of prepediocin to pediocin AcH in *Pediococcus acidilactici *H. Annu Meeting 1992, New Orleans, LA, May 26–30 Abstr 081.

[B34] Henderson JT, Chopco AL, van Wassenaar PD (1991). Purification and primary structure of pediocin PA-1 produced by *Pediococcus acidilactici *PAC1 0. Arch Biochem Biophys.

[B35] Lozano JCN, Meyer JN, Sletten K, Pelaz C, Ness IF (1992). Purification and amino acid sequence of a bacteriocin produced by *Pediococcus acidilactici*. J Gen Microbiol.

[B36] Anastasiadou S, Papagianni M, Filiousis G, Ambrosiadis I, Koidis P (2008). Pediocin SA-1, an antimicrobial peptide from *Pediococcus acidilactici *NRRL B5627: Production conditions, purification and characterization. Biores Technol.

[B37] Sire JM, Donnio PY, Mesnard R, Pouëdras P, Avril JL (1992). Septicemia and hepatic abscess caused by *Pediococcus acidilactici*. Eur J Clin Microbiol Infect Dis.

[B38] Dobrogosz WJ, Stone RW (1962). Oxidative metabolism in *Pediococcus pentosaceus*. I. Role of oxygen and catalase. J Bacteriol.

[B39] Delwiche EA (1961). Catalase of *Pediococcus cerevisiae*. J Bacteriol.

[B40] Raccach M (1987). *Pediococci *and biotechnology. Crit Rev Microbiol.

[B41] Kimura H, Nagano R, Matsusaki H, Sonomoto K, Ishizaki A (1997). A bacteriocin of strain *Pediococcus *sp. ISK-1 isolated from Nukadoko, bed of fermented rice bran. Biosci Biotechnol Biochem.

[B42] Makarova K, Slesarev A, Wolf Y, Sorokin A, Mirkin B, Koonin E, Pavolv A, Pavlova N, Karamychev V, Polouchine N, Shakhova V, Grigoriev I, Lou Y, Rohksar D, Lucas S, Huang K, Goldstein DM, Hawkins T, Plengvidhya V (2006). Comparative genomics of the lactic acid bacteria. Proc Natl Acad Sci USA.

[B43] Entrez Genome Project Pediococcus pentosaceus ATCC 25745.

[B44] Site. http://www.membranetransport.org.

[B45] Corcoran GD, Gibbons N, Mulvihill TE (1991). Septicemia caused by *Pediococcus pentosaceus*: a new opportunistic pathogen. J Infect.

[B46] Barton LL, Ryder ED, Cohen RW (2001). Bacteremic Infection with *Pediococcus*: vancomycin-resistant opportunist. Pediatrics.

[B47] Collins MD, Rodrigues UM, Ash C, Aguirre M, Farrow JAE, Martinez-Murcia A (1991). Phylogenic analysis of the genus *Lactobacillus *and related lactic acid bacteria determined by reverse transcriptase sequencing of 16S rRNA. FEMS Microbiol Lett.

[B48] Suzuki K, Ozaki K, Yamashita Y (2004). Comparative analysis of conserved genetic markers and adjacent DNA regions identified in beer-spoilage lactic acid bacteria. Lett Appl Microbiol.

[B49] Suzuki K, Sami M, Iijima K, Ozaki K, Yamashita Y (2006). Characterization of horA and its flanking regions of *Pediococcus damnosus *ABBC478 and development of more specific and sensitive horA PCR method. Lett Appl Microbiol.

[B50] Calmin G, Lefort L, Belbahri L (2008). Multi-loci sequence typing (MLST) for two lacto-acid bacteria (LAB) species: *Pediococcus parvulus *and *P. damnosus*. Mol Biotechnol.

[B51] Li A, Kong F (2004). Synthesis of an alpha-linked dimmer of the trisaccharide repeating unit of the exopolysaccharide produced by *Pediococcus damnosus *2.6. Carbohydr Res.

[B52] Skytta E, Haikara A, Mattila-Sandholm T (1993). Production and characterization of antibacterial compounds produced by *Pediococcus damnosus *and *Pediococcus pentosaceus*. J Appl Bacteriol.

[B53] Nel HA, Bauer R, Vandamme EJ, Dicks LM (2001). Growth optimization of *Pediococcus damnosus *NCFB1832 and the influence of pH and nutrients on the production of pediocin PD-1. J Appl Microbiol.

[B54] Bauer R, Chikindas ML, Dicks LMT (2005). Purification, partial amino acid sequence and mode of action of pediocin PD-1, a bacteriocin produced by *Pediococcus damnosus *NCFB1832. Int J Food Microbiol.

[B55] Biswas SR, Ray P, Johnson MC, Ray B (1991). Influence of growth conditions on the production of a bacteriocin, pediocin AcH, by *Pediococcus acidilactici *H. Appl Environ Microbiol.

[B56] Anastasiadou S, Papagianni M, Filiousis G, Ambrosiadis I, Koidis P (2008). Growth and metabolism of a meat isolated strain of *Pediococcus pentosaceus *in submerged fermentation. Purification, characterization and properties of the produced pediocin SM-1. Enzyme MicrobTechnol.

[B57] Mundt JR, Beattie WG, Wieland FR (1969). *Pediococci *residing in plants. J Bacteriol.

[B58] Vazquez JA, Gonzalez MP, Murado MA (2003). Substrate inhibition of *Pediococcus acidilactici *by glucose on a waste medium. Simulations and experimental results. Lett Appl Microbiol.

[B59] Kim WJ, Ray B, Johnson MC (1992). Plasmid transfers by conjugation and electroporation in *Pediococcus acidilactici*. J Appl Bacteriol.

[B60] Altay G, Bozoglu F, Ray B (1994). Efficiency of gene transfer by conjugation and electroporation in lactococci and pediococci. Food Microbiol.

[B61] Caldwell S, Hutkins RW, McMahon DJ, Oberg CJ, Broadbent JR (1998). Lactose and galactose uptake by genetically engineered *Pediococcus *species. Appl Microbiol Biotechnol.

[B62] Barthelmebs L, Lecomte B, Divies C, Cavin JF (2000). Inducible metabolism of phenolic acids in *Pediococcus pentosaceus *is encoded by an autoregulated operon which involves a new class of negative transcriptional regulator. J Bacteriol.

[B63] Mora D, Musacchio F, Fortina MG, Senini L, Manachini PL (2003). Autolytic activity and pediocin-induced lysis in *Pediococcus acidilactici *and *Pediococcus pentosaceus *strains. J Appl Microbiol.

[B64] Ray B, Ray B, Daeschel M (1992). Pediocin(s) of *Pediococcus acidilactici *as a food biopreservative. Food biopreservatives of microbial origin.

[B65] Anastasiadou S, Papagianni M, Ambrosiadis I, Koidis P (2008). Rapid quantifiable assessment of nutritional parameters influencing pediocin production by *Pediococcus acidilactici *NRRL B5627. Biores Technol.

[B66] Cabo ML, Murado MA, Gonzales MP, Pastoriza L (2001). Effects of aeration and pH gradient on nisin production. A mathematical model. Enzyme Microb Technol.

[B67] Amiali MN, Lacroix C, Simard RE (1998). High nisin Z production by *Lactococcus lactis *UL719 in whey permeate with aeration. World J Microbiol Biotechnol.

[B68] De Vuyst L, Callewaert R, Crabbe K (1996). Primary metabolite kinetics of bacteriocin biosynthesis by *Lactobacillus amylovorus *and evidence for stimulation of bacteriocin production under unfavourable growth conditions. Microbiology.

[B69] Osmanagaoglou O, Gunduz U, Beyatli Y, Cokmus C (1998). Purification and characterization of pediocin F, a bacteriocin produced by *Pediococcus acidilactici *F. Tr J of Biol.

[B70] Wu CW, Yin LJ, Jiang ST (2004). Purification and characterization of bacteriocin from *Pediococcus pentosaceus *ACCEL. J Agr Food Chem.

[B71] Yin LJ, Wu CW, Jiang ST (2003). Bacteriocins from *Pediococcus pentosaceus *L and S from pork meat. J Agr Food Chem.

[B72] Osmanagaoglou O, Beyatli Y, Gunduz U (2001). Isolation and characterization of pediocin producing *Pediococcus pentosaceus *Pep1 from vacuum-packed sausages. Turk J Biol.

[B73] Todorov SD, Dicks LMT (2005). Pediocin ST18, an antilisterial bacteriocin produced by *Pediococcus pentosaceus *ST18 isolated from boza, a traditional cereal beverage from Bulgaria. Process Biochem.

[B74] Guerra NP, Pastrana L (2002). Dynamics of pediocin biosynthesis in batch fermentation on whey. Electron J Environ Agric Food Chem.

[B75] Guerra NP, Pastrana L (2002). Modelling the influence of pH on the kinetics of both nisin and pediocin production and characterization of their functional properties. Process Biochem.

[B76] Guerra NP, Pastrana L (2003). Influence of pH drop on both nisin and pediocin production by *Lactococcus lactis *and *Pediococcus acidilactici*. Lett Appl Microbiol.

[B77] Vazquez JA, Gonzalez MP, Murado MA (2004). Nisin and pediocin production by *Lactococcus lactis *and *Pediococcus acidilactici *using waste protein sources from octopus. Electron J Environ Agric Food Chem.

[B78] Nel HA, Vandamme EJ, Dicks LMT (2001). Growth optimization of *Pediococcus damnosus *NCFB 1832 and the influence of pH and nutrients on the production of pediocin PD-1. J Appl Microbiol.

[B79] Guerra NP, Pastrana L (2002). Nisin and pediocin production on mussel-processing waste supplemented with glucose and five nitrogen sources. Lett Appl Microbiol.

[B80] Vazquez JA, Gonzalez MP, Murado MA (2004). Peptones from autohydrolysed fish viscera for nisin and pediocin production. J Biotechnol.

[B81] Guerra NP, Bernardez PF, Agrasar AT, Macias CL, Pastrana L (2005). Fed-batch pediocin production by *Pediococcus acidilactici *NRRL-B5627 on whey. Biotechnol Appl Biochem.

[B82] Guerra NP, Agrasar AT, Macias CL, Pastrana L (2005). Modelling the fed-batch production of pediocin using mussel processing wastes. Process Biochem.

[B83] Guerra NP, Bernardez PF, Pastrana L (2008). Modelling the stress inducing biphasic growth and pediocin production by *Pediococcus acidilactici *NRRL-B5627 in re-alkalized fed-batch cultures. Biochem Eng J.

[B84] Cho HY, Yousef AE, Yang ST (1996). Continuous production of pediocin by immobilized *Pediococcus acidilactici *PO2 in a packed-bed bioreactor. Appl Microbiol Biotechnol.

[B85] Liao CC, Yousef AE, Richter ER, Chism GW (1993). *Pedioccus acidilactici *PO2 bacteriocin production in whey permeate and inhibition of *Listeria monocytogenes *in foods. J Food Sci.

[B86] Huang J, Lacroix C, Daba H, Simard RE (1996). Pediocin production and plasmid stability during continuous free and immobilized cell cultures of *Pediococcus acidilactici *UL5. J Appl Bacteriol.

[B87] Naghmouchi K, Fliss I, Drider D, Lacroix C (2008). Pediocin PA-1 production during repeated-cycle batch culture of immobilized *Pediococcus acidilactici *UL5 cells. J Biosci Bioeng.

[B88] Vazquez Alvarez JA, Gonzalez MP, Murado MA (2004). Pediocin production by *Pediococcus acidilactici *in solid state culture on a waste medium: Process simulation and experimental results. Biotechnol Bioeng.

[B89] Drider D, Fimland G, Hechard Y, McMullen LM, Prevost H (2006). The continuing story of class IIa bacteriocins. Microbiol Mol Biol Rev.

[B90] Graham DC, McKay LL (1985). Plasmid DNA in strains of *Pediococcus cerevisia*e and *Pediococcus pentosaceus*. Appl Environ Microbiol.

[B91] Daeschel MA, Klaenhammer TR (1985). Association of a 13.6 MDal plasmid in *Pediococcus pentosaceus *with bacteriocin activity. Appl Environ Microbiol.

[B92] Hoover DG, Walsh PM, Kolaetis KM, Daly MM (1988). A bacteriocin produced by *Pediococcus *species associated with a 5.5 kDal plasmid. J Food Prot.

[B93] Gonzalez CF, Kunka BS (1987). Plasmid-associated bacteriocin production and sucrose fermentation in *Pediococcus acidilactici*. Appl Environ Microbiol.

[B94] Ray SK, Johnson MC, Ray B (1989). Bacteriocin plasmids in *Pediococcus acidilactici*. J Ind Microbiol.

[B95] Ennahar S, Sashihara T, Sonomoto K, Ishizaki A (2000). Class IIa bacteriocins: biosynthesis, structure and activity. FEMS Microbiol Rev.

[B96] Miller KW, Schamber R, Osmanagaoglou O, Ray B (1998). Isolation and characterization of pediocin AcH chimeric protein mutants with altered bactericidal activity. Appl Environ Microbiol.

[B97] Quadri LE, Yan LZ, Stiles ME, Vederas JC (1997). Effect of amino acid substitutions on the activity of carnobacteriocin B2. Overproduction of the antimicrobial peptide, its engineered variables and its precursor in *Escherichia coli*. J Biol Chem.

[B98] Chen Y, Ludescher RD, Montville TJ (1997). Electrostatic interactions but not the YGNGV consensus motif, govern the binding of pediocin PA-1 and its fragments to phospholipids vesicles. Appl Environ Microbiol.

[B99] Kazazic M, Nissen-Meyer J, Fimland G (2002). Mutational analysis of the role of charged residues in target-cell binding, potency and specificity of the pediocin-like bacteriocin sakacin P. Microbiology.

[B100] Uteng M, Hauge HH, Markwick PR, Fimland G, Mantzilas D, Nissen-Meyer J, Muhle-Goll C (2003). Three-dimensional structure in lipid micelles of the pediocin-like antimicrobial peptide sakacin P and a sakacin P variant that is structurally stabilized by an inserted C-terminal disulphide bridge. Biochemistry.

[B101] Fimland G, Blingsmo OR, Sletten K, Jung G, Nes IF, Nissen-Meyer J (1996). New biologically active hybrid bacteriocins constructed by combining regions from various pediocin-like bacteriocins: the C-terminal region is important for determining specificity. Appl Environ Microbiol.

[B102] Fimland G, Jack R, Jung G, Jung G, Nes IF, Nissen-Meyer J (1998). The bactericidal activity of pediocin PA-1 is specifically inhibited by a 15-mer fragment that spans the bacteriocin from the center toward the C terminus. Appl Environ Microbiol.

[B103] Cintas LM, Rodriguez JM, Fernandez MF, Sletten K, Nes IF, Hernandez PE, Holo H (1995). Isolation and characterization of pediocin L50, a new bacteriocin from *Pediococcus acidilactici *with a broad inhibitory spectrum. Appl Environ Microbiol.

[B104] Elegado FB, Kin WJ, Kwon DY (1997). Rapid purification, partial characterization, and antimicrobial spectrum of the bacteriocin, pediocin AcM, from *Pediococcus acidilactici *M. Int J Food Microbiol.

[B105] Schved F, Lalazar Y, Henis Y, Juven BJ (1993). Purification, partial characterization and plasmid-linkage of pediocin SJ-1, a bacteriocin produced by *Pediococcus acidilactici*. J Appl Bacteriol.

[B106] Strässer de Saad AM, Pasteris SE, Manca de Nadra MC (1995). Production and stability of pediocin N5p in grape juice medium. J Appl Bacteriol.

[B107] Shin MS, Han SK, Ryu JS, Kim KS, Lee WK (2008). Isolation and partial characterization of a bacteriocin produced by *Pediococcus. pentosaceus *K23-2 isolated from kimchi. J Appl Microbiol.

[B108] Eijsink VG, Sheie M, Middelhoven PH, Brurberg MB, Nes IF (1998). Comparative studies of class IIa bacteriocins of lactic acid bacteria. Appl Environ Microbiol.

[B109] Fimland G, Johnsen L, Axelsson L, Brurberg MB, Nes IF, Eijsink VGH, Nissen-Meyer J (2000). A C-terminal disulfide bridge in pediocin-like bacteriocins renders bacteriocin activity less temperature dependent and is a major determinant of the antimicrobial spectrum. J Bacteriol.

[B110] Bhunia AK, Johnson MC, Ray B, Kalchayanad N (1991). Mode of action of pediocin AcH from *Pediococcus acidilactici *H on sensitive bacterial strains. J Appl Bacteriol.

[B111] Motlagh AM, Johnson MC, Ray B (1991). Viability loss of food borne pathogens by starter culture metabolites. J Food Prot.

[B112] Moll GN, Konings WN, Driessen AJM (1999). Bacteriocins: mechanism of membrane insertion and pore formation. Antonie van Leeuwenhoek.

[B113] Derksen DJ, Boudreau MA, Vederas JC (2008). Hydrophobic interactions as substitutes for a conserved disulfide linkage in the type IIa bacteriocins, leucocin A and pediocin PA-1. Chem Bio Chem.

[B114] Kaur K, Andrew LC, Wishart DS, Vederas JC (2004). Dynamic relationships among type IIa bacteriocins: temperature effects on antimicrobial activity and on structure of the C-terminal amphipathic α helix as a receptor-binding region. Biochemistry.

[B115] Deegan LH, Cotter PD, Hill C, Ross P (2006). Bacteriocins: biological tools for bio-preservation and shelf-life extension. Int Dairy J.

[B116] Bhowmik T, Marth EH (1990). β-galactosidase of *Pediococcus *species: induction, purification and partial characterization. Appl Microbiol Biotechnol.

[B117] Guinane CM, Cotter PD, Hill C, Ross RP (2005). Microbial solutions to microbial problems: Lactococcal bacteriocins for the control of undesirable biota in food. J Appl Microbiol.

[B118] Somkuti GA, Steiberg DH (2003). Pediocin production by recombinant lactic acid bacteria. Biotechnol Lett.

[B119] Brumfitt W, Salton MRJ, Hamilton-Miller JMT (2002). Nisin alone and combined with peptidoglycan-moduling antibiotics: activity against methicillin-resistant *Staphylococcus aureus *and vancomycin-resistant enterococci. J Anticrobiol Chemother.

[B120] Giacometti A, Cirioni O, Barchiesi F, Fortuna M, Scalise G (1999). In-vitro activity of cationic peptides alone and in combination with clinically used antimicrobial agents against *Pseudomonas aeruginosa*. J Anticrobiol Chemother.

[B121] Wu J, Hu S, Cao L (2007). Therapeutic effect of nisin Z on subclinical mastitis in lactating Cows. Antimicrob Agents Chemother.

